# Suppression of the Macrophage Proteasome by Ethanol Impairs MHC Class I Antigen Processing and Presentation

**DOI:** 10.1371/journal.pone.0056890

**Published:** 2013-02-25

**Authors:** Alain J. D’Souza, Shyamal D. Desai, Xiaowen L. Rudner, Michelle N. Kelly, SanBao Ruan, Judd E. Shellito

**Affiliations:** 1 Department of Genetics, Louisiana State University Health Sciences Center, New Orleans, Louisiana, United States of America; 2 Department of Biochemistry and Molecular Biology, Louisiana State University Health Sciences Center, New Orleans, Louisiana, United States of America; 3 Section of Pulmonary/Critical Care Medicine – Department of Medicine, Louisiana State University Health Sciences Center, New Orleans, Louisiana, United States of America; Tulane University, United States of America

## Abstract

Alcohol binge-drinking (acute ethanol consumption) is immunosuppressive and alters both the innate and adaptive arms of the immune system. Antigen presentation by macrophages (and other antigen presenting cells) represents an important function of the innate immune system that, in part, determines the outcome of the host immune response. Ethanol has been shown to suppress antigen presentation in antigen presenting cells though mechanisms of this impairment are not well understood. The constitutive and immunoproteasomes are important components of the cellular proteolytic machinery responsible for the initial steps critical to the generation of MHC Class I peptides for antigen presentation. In this study, we used an *in-vitro* cell culture model of acute alcohol exposure to study the effect of ethanol on the proteasome function in RAW 264.7 cells. Additionally, primary murine peritoneal macrophages obtained by peritoneal lavage from C57BL/6 mice were used to confirm our cell culture findings. We demonstrate that ethanol impairs proteasome function in peritoneal macrophages through suppression of chymotrypsin-like (Cht-L) proteasome activity as well as composition of the immunoproteasome subunit LMP7. Using primary murine peritoneal macrophages, we have further demonstrated that, ethanol-induced impairment of the proteasome function suppresses processing of antigenic proteins and peptides by the macrophage and in turn suppresses the presentation of these antigens to cells of adaptive immunity. The results of this study provide an important mechanism to explain the immunosuppressive effects of acute ethanol exposure.

## Introduction

Alcohol abuse is a major health concern in the United States. Episodic excessive (acute) alcohol consumption or binge drinking accounts for 23% of the alcohol abusers in the United States [1]. Alcohol binge-drinking, defined as more than five drinks of ethanol consumed over a short time period, is immunosuppressive [2], [3]. Binge drinking alters and suppresses cellular functions of both the innate and adaptive arms of the immune system including macrophage functions such as phagocytosis, cytokine and chemokine release, and antigen presentation [4]–[6]. However, molecular mechanisms by which ethanol induces suppression of the immune response are poorly understood and not well defined.

The two ‘arms’ of the immune system are the innate and the adaptive immune systems. The innate immune system is the first line of defense during the host immune response. Antigen presenting cells (APC’s) such as macrophages and dendritic cells play an important role in the innate immune response. By efficiently processing and presenting self and non-self-proteins to T-lymphocytes these APC’s orchestrate an efficient immune response within the host. The ability of these cells to effectively process foreign antigens for presentation is crucial in determining how rapidly the adaptive immune system responds and, in concert with innate immunity, efficiently mounts an immune response [7], [8].

Antigen processing involves protein fragmentation (proteolysis) and loading of the peptide fragments onto the MHC (major histocompatibility complex) molecules for their presentation to T cells. In macrophages, as well as other APC’s, a large number of the protein fragments required for MHC Class I presentation are generated by the proteasome [9]–[11]. Proteasomes are intracellular, multi-subunit catalytic proteases responsible for the protein turnover within the cell [12], [13]. Most cells express the 26S proteasome, which is composed of a constitutive 20S (c20S) catalytic core protease, capped by the 19S regulatory complex at each end [12], [13]. Constitutively expressed mammalian 20S proteasomes have three active subunits, beta1, beta 2, and beta 5, possessing post-glutamyl peptide hydrolase-like (PGPH) (*i.e.* caspase-like (C-L), trypsin-like (T-L), and chymotrypsin-like (Cht-L) activities respectively [14]. These subunits are responsible for cleaving proteins into short, 3–22 amino acid long, polypeptides [15], [16]. In hematopoietic cells and cells stimulated with the cytokine interferon gamma (IFN-gamma), a variant form of the proteasome, the immunoproteasome (i20S), is expressed in addition to the constitutive 20S proteasome [15], [16]. In the immunoproteasome, the beta1, beta 2, and beta 5 catalytic subunits of the c20S are replaced by immunoproteasome subunits Low Molecular Mass Polypeptide 2 (LMP2) (beta1i), Multicatalytic Endopeptidase Complex Subunit (MECL-1) (beta 2i) and Low Molecular Mass Polypeptide 7 (LMP7) (beta 5i) respectively [15], [16]. These subunits, along with the PA28 regulatory subunit, confer altered substrate specificity to the i20S [15], [16]. The constitutive and immunoproteasomes are vital in generating antigenic peptides for MHC Class I antigen presentation [10], [17], as proteasome inhibitors that blocked the degradation of the cellular proteins, also blocked the antigen presentation in a number of different cell types [18], [19].

Prior work has demonstrated that ethanol suppresses proteasome activity and antigen presentation in hepatocytes [20], [21]. In this study we demonstrate that ethanol suppresses proteasome function in peritoneal macrophages. This suppression leads to impaired processing and presentation of ovalbumin and C-terminal extended ovalbumin peptide SIINFEKLTE in a MHC Class I restricted context by the macrophages to cytotoxic T lymphocytes (CD8+ T cells). Our studies thus reveal the inhibition of the proteasome-mediated antigen presentation as an important mechanism underlying the immunosuppressive effects of alcohol.

## Materials and Methods

### Ethics Statement

Animal study was carried out in strict accordance with the recommendations in the Guide for the Care and Use of Laboratory Animals of the National Institutes of Health. The protocol was approved by the Louisiana State University Health Sciences Center (LSUHSC) - New Orleans Institutional Animal Care and Use Committee under its assurance (A3094-01) with the Office of Laboratory Animal Welfare of the National Institutes of Health. All surgery was performed under ketamine/xylazine anesthesia and all effort was made to minimize suffering to the animal. Mice were administered intramuscular ketamine/xylazine (200 mg/kg/10 mg/kg) solution and anesthesia was determined by toe pinch reflex. Wherever possible, animals were euthanized by rapid cervical dislocation following anesthesia.

### Cell Lines

The murine macrophage/monocyte cell line, RAW 264.7 (TIB-71), was obtained from the American Type Culture Collection (ATCC), Manassas, VA. RAW 264.7 cells were maintained in high glucose Dulbecco’s Modified Eagle’s Medium (DMEM) with pyruvate (Invitrogen, Carlsbad, CA) supplemented with 10% heat-inactivated HyClone fetal bovine serum (Thermo Scientific, Rockford, IL) and 1% penicillin-streptomycin (Invitrogen, Carlsbad, CA) in a 5% CO_2_ 37°C incubator.

### Animals

Specific pathogen-free C57BL/6 mice (MHC haplotype H2^b^) (9 weeks old) were purchased from NCI/Charles River Breeding Laboratories (Wilmington, MA). Specific pathogen-free OT-1 transgenic mice [C57BL/6-Tg (TcraTcrb) 1100Mjb/J; MHC Haplotype H2-K^b^; 10 weeks old] were purchased from the Jackson Laboratory (Bar Harbor, ME). Animals were allowed to recover from the stress of transit for 5 days before any procedure was performed on them. All mice were housed in specific pathogen-free rooms in filter-topped cages within the animal care facilities of the Louisiana State University Health Sciences. Mice were provided with water and food *ad libitum* and received 12-h light/dark cycles.

### Isolation of Peritoneal Macrophages

Naïve C57BL/6 mice were anesthetized with ketamine/xylazine and then euthanized by rapid cervical dislocation. Using sterile dissection techniques, the skin from the abdominal cavity was opened to reveal the peritoneal cavity. With a 22.5G needle attached to a syringe, 10 ml warm PBS (Invitrogen, Carlsbad, CA) was introduced into the peritoneal cavity. The cavity was gently palpated and the fluid withdrawn into the syringe and dispensed into a 50 ml conical tube and stored on ice. Lavaged peritoneal cells from multiple mice were pooled together. The cells were spun down at 400×g, resuspended in DMEM/F12 medium (Invitrogen, Carlsbad, CA) supplemented with 10% heat-inactivated fetal bovine serum, and 1% penicillin-streptomycin (Complete DMEM/F12-10 medium). Live cell number was determined by trypan blue and hemocytometer counting. For experiments, 2.5×10^5^ peritoneal macrophages were seeded into 48 well tissue culture treated plates (Corning, Lowell, MA) and were adherence enriched for 1.5 h. Unattached cells were aspirated and discarded, attached macrophages washed with PBS, replenished with complete medium and placed overnight in a 5% CO_2_ 37°C incubator before proceeding to experimental manipulations.

### Isolation of CD8+T Lymphocytes

CD8+ T lymphocytes were isolated, by negative selection, from the spleen of OT-1 mice using the CD8a+ T Cell Isolation Kit II (Miltenyi Biotec, Auburn, CA). In brief, single-cell suspensions of splenocytes were prepared by mechanically disrupting the tissue, in the manufacturer recommended isolation buffer, with a sterile syringe plunger through a 40 um nylon cell strainer (BD Biosciences, Bedford, MA). Red cells were lysed using red cell lysis buffer (Sigma, St. Louis, MO). Cells were centrifuged at 400×g and resuspended in isolation buffer. Magnetic labeling of non CD8+ T cells was carried out as recommended by the manufacturer. The labeled cells were then passed through a magnetic column and the effluent cells were collected as enriched CD8+ T lymphocytes. Typical purity of isolated CD8+ T lymphocytes was about 90 percent and was confirmed by flow cytometry (data not shown). Enriched cells were pelleted and resuspended in complete DMEM/F12-10 medium to the required cell concentration (6.25×10^5^ cells/ml).

### Ethanol Exposure, Inhibitor Treatments and Bacterial Challenge

RAW 264.7 cells (2×10^5^ cell/ml) or primary peritoneal macrophages (2.5×10^5^ cells) were exposed to ethanol (200 proof) (Pharmco-Aaper, Brookfield, CT) by replacing the culture medium with ethanol-containing culture medium at the indicated concentrations (50 mM or 100 mM) for the time periods indicated in the experiments. Ethanol-treated cultures were kept in 5% CO_2_ 37°C incubators that had been pre-saturated with the appropriate ethanol concentration. Control (0 mM) cultures were maintained in complete medium in normal 5% CO_2_ 37°C incubators. Wherever indicated, cells were treated with the proteasome inhibitors, InSolution™ MG132 (Carbobenzoxy-L-leucyl-L-leucyl-L-leucinal, Z-LLL-CHO) (EMD Chemicals, Gibbstown, NJ) (0.5 uM or 5 uM) or Lactacystin (Millipore, Billerica, MA) (10 uM) to inhibit proteasome function. *Klebsiella pneumoniae* (*Kp*) strain 43816 serotype 2 (ATCC, Manassas, VA) was cultured in 100 ml Luria-Bertani (LB) medium on an orbital shaker (180 rpm) at 37°C for 18 h. Bacterial cells were pelleted by centrifugation, washed 2X times with sterile PBS, serially diluted in sterile PBS and plated out on McConkey agar plates to determine initial viable culture concentration. Aliquots of the culture were heat-killed on a 70°C water bath for 2 hours. An aliquot of this heat-killed stock was plated out to ensure that none of the culture was viable. Ethanol-treated macrophages were challenged with the heat- killed *K. pneumoniae* at a multiplicity of infection (M.O.I.) of 10∶1. *Kp* challenge was carried out 6 hours post ethanol exposure for various times as indicated.

### 20S Proteasome Activity Assay

The proteolytic chymotrypsin-like activity of the proteasome was evaluated in whole cell lysates using a 20S proteasome activity assay kit (Millipore, Billerica, MA), as described by the manufacturer. In brief, ethanol-exposed unstimulated and *Kp*-stimulated macrophages were lysed using ice cold lysis buffer (50 mM HEPES pH 7.5, 5 mM EDTA, 150 mM NaCl, 2 mM ATP, 1% TritonX-100), sonicated and centrifuged. Total protein levels in the clear cell lysates were determined using a BCA protein assay kit (Thermo Scientific, Rockford, IL). 20 ug total protein was incubated in the provided buffer with ﬂuorophore-labeled peptide substrate (LLVY-7-amino-4-methylcoumarin [AMC]) for 120 min at 37°C. Proteasome activity was measured using a 380/460 nm filter set in a ﬂuorometer (Synergy HT multi-mode microplate reader, BioTek, Winooski, VT) by quantification of the free AMC released by proteasome cleavage of peptide substrate and is represented as relative ﬂuorescent units (R.F.U.). The purified 20S proteasome subunit and the proteasome inhibitor lactacystin (provided by the manufacturer) were used as controls for the assay.

### Immunoblot Analysis of Macrophage Proteins

RAW 264.7 cells (2×10^5^ cells/ml) were treated with ethanol for the indicated time points as described before. Cells were then lysed using SDS lysis buffer (2% SDS. 50 mM Tris-HCl pH 7.5), sonicated, centrifuged, and boiled for 10 mins to denature the proteins. Protein concentration of clarified lysates was determined by BCA protein assay kit (Thermo Scientific, Rockford, IL). Protein levels were normalized in 2× SDS loading buffer. 5 ug of total protein was resolved on a 15% SDS-PAGE gel (29∶1) (Bio-Rad Mini Protean™ system). Samples were then transferred to Immun-Blot PVDF membranes (Bio-Rad, Hercules, CA) and blocked for one hour in 5% non-fat milk at room temperature. Immunodetection was carried out overnight at 4°C, with primary rabbit anti-mouse antibodies specific for PSMB5 (Abcam, Cambridge, MA), or LMP7 (Abcam, Cambridge, MA) at 1∶1000 dilution (1 µg/ml). To confirm equal protein loading, each blot was stripped with Restore Western *Blot* Stripping Buffer (Thermo Scientific, Rockford, IL) and reprobed with anti-mouse beta-actin (1∶10,000) (100 ng/ml) (Abcam, Cambridge, MA). Secondary goat anti-rabbit HRP-conjugated antibody (Abcam, Cambridge, MA) and horse anti-mouse HRP-conjugated antibody (Cell Signaling Technology, Danvers, MA) were used at 1∶10,000 dilution followed by visualization by enhanced chemiluminescence using SuperSignal West Dura Extended Duration Substrate (Thermo Scientific, Rockford, IL). Protein levels were quantified by densitometry using Quantity One Software (BioRad, Hercules, CA).

### Antigen Processing and Presentation

C-terminal extended SIINFEKL peptide (SIINFEKLTE) was custom synthesized by GenScript (GenScript, Piscataway, NJ). Whole length chicken ovalbumin (Sigma, St. Louis, MO) and SIINFEKLTE were prepared at the required concentration in complete DMEM F12/10 medium, aliquoted and stored at −20°C. Primary peritoneal macrophages (2.5×10^5^ cells/well) were exposed to 50 mM or 100 mM ethanol in complete DMEM/F12-10 medium, as described earlier, for 24, 48 or 72 hours. Primary peritoneal macrophages were also treated with the proteasome inhibitors MG132 (5 uM) and Lactacystin (10 uM) as positive controls. Cells were exposed to whole length chicken ovalbumin (2 mg/ml) or SIINFEKLTE (10 ug/ml) 2 hours prior to co-incubation with CD8+ T lymphocytes. The macrophages were washed 3 times with sterile PBS to remove ethanol and soluble antigen and overlaid with enriched CD8+ T cells from OT-1 mouse spleen at a ratio of 2∶1 (i.e. 1.25×10^5^ CD8+ T cells/well) in complete DMEM/F12-10 medium. The co-incubation was carried out for 18 h after which cell supernatants were collected and analyzed for levels of cytokine Interleukin 2 (IL-2) (pg/ml) using Quantikine Mouse IL-2 ELISA kit (R&D Systems, Minneapolis, MN) as recommended by the manufacturer.

### Statistical Analysis

Paired Student’s t-test was used to determine if there were significant differences between experimental mean values of the various groups. A p-value of ≤0.05 was considered statistically significant. All statistical analysis was performed using GraphPad Prism Software (GraphPad Software, Inc. La Jolla, CA) and graphs plotted in Microsoft Excel (Microsoft, Inc. Redmond, WA).

## Results

### Ethanol Suppresses the 20S Chymotrypsin-like (Cht-L) Proteasome Activity in RAW 264.7 Cells

**Figure 1:**
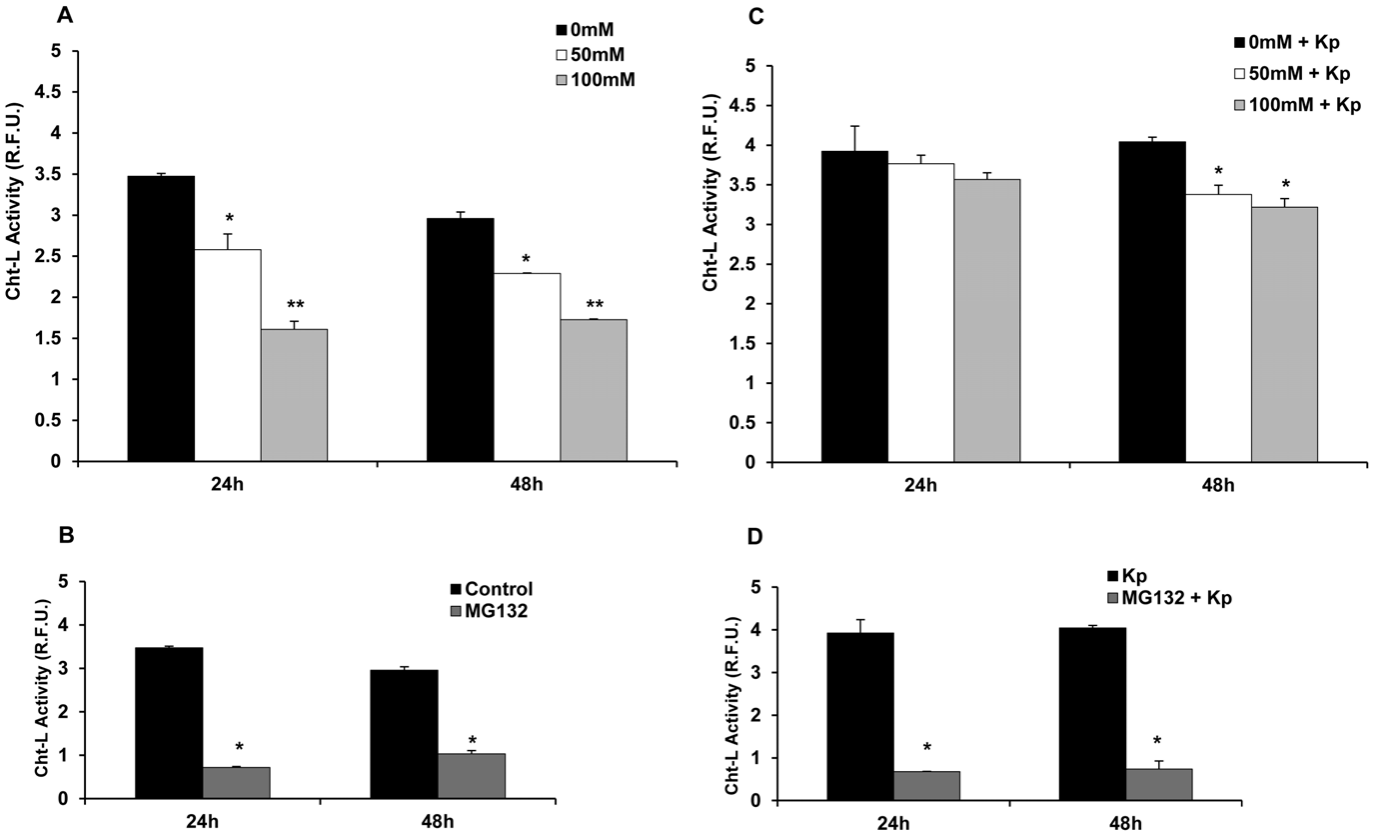
20S chymotrypsin-like (Cht-L) proteasome activity is suppressed in ethanol-treated RAW 264.7 cells. A. Murine RAW 264.7 (2×10^5^ cells/ml) cells were treated with 50 mM or 100 mM ethanol (EtOH) for 24 h or 48 h. 0 mM (control) cells received no treatment. Cell lysates obtained at the indicated time points were analyzed for 20S Cht-L proteasome activity by fluorigenic assay as described in Methods. **B.** RAW 264.7 cells were treated with MG132 (0.5 uM) as a positive control of proteasome suppression. Cell lysates were analyzed at the indicated time points for proteasome activity as described in panel A. **C.** Ethanol-treated RAW 264.7 cells were stimulated with heat-killed *K.pneumoniae* (*Kp*), 6 hours after initial EtOH exposure. Cell lysates obtained at the indicated time points were analyzed as described above in panel A. **D.** RAW 264.7 cells were treated with MG132 (0.5 uM) followed by challenge with *Kp* as described in panel C. Cells were lysed at the indicated time points and analyzed for proteasome activity as described above. Proteasome activity data was plotted as mean relative fluorescence units (R.F.U.) ± SEM (three replicates per experiment) and is representative of three independent experiments. Higher R.F.U. values indicate higher proteasome activity. *p<0.05 vs. control (0 mM) at the respective time point. **p<0.05 vs. control (0 mM) and 50 mM EtOH at the respective time point.

Proteasome is important cellular proteolytic machinery responsible for regulation of intracellular protein turnover [22]. Impairment of proteasome function has vast effects on cellular function and homeostasis [23]. To assess the effect of acute ethanol exposure on proteasome activity in macrophages, RAW 264.7 cells were treated with 50 mM or 100 mM ethanol for either 24 or 48 hours. Control (0 mM) cells received no treatment. Viability of the macrophages was unaffected in the ethanol-treated groups *vs*. the respective controls as confirmed by trypan blue staining and microscopy (Data not shown). At the indicated time points, the cells were lysed and analyzed for 20S Cht-L proteasome activity as described in the Methods Section. As shown in Fig. 1A, ethanol suppressed proteasome activity in a dose-dependent manner in RAW 264.7 cells. (Higher R.F.U. values indicate higher proteasome activity). Proteasome activity was significantly decreased to 26% (R.F.U. 2.58±0.192) and 54% (R.F.U. 1.61±0.098) in RAW 264.7 cells exposed to 50 mM or 100 mM EtOH respectively for 24 h as compared to non-treated (0 mM) RAW 264.7 cells (R.F.U. 3.47±0.034). A similar dose-dependent suppression of proteasome activity was also observed in 48 h ethanol-treated RAW 264.7 cells as compared to the respective control. Under identical assay conditions, MG132 (0.5 uM), a well-established proteasome inhibitor [13], [22], significantly suppressed proteasome activity up to 79% (R.F.U. 0.72±0.024) and 65% (R.F.U. 1.03±0.078) at 24 h and 48 h respectively as compared to the untreated controls at those time points (Fig. 1B), thus confirming the validity of the proteasome assay conditions.

We also examined the effect of acute ethanol exposure on 20S Cht-L proteasome activity in stimulated macrophages; a macrophage phenotype relevant to the host response. Ethanol-treated RAW 264.7 cells were stimulated with heat-killed, gram-negative bacterium, *Klebsiella pneumoniae* (*Kp*) (MOI 10∶1) as described in the Methods section. Viability of the stimulated macrophages remained unaffected in the ethanol-treated *Kp*-stimulated groups *vs*. the respective *Kp*-stimulated group as confirmed by trypan blue staining and microscopy (Data not shown). Analysis of the 24 h cell lysates showed a significant suppression in proteasome activity of the 50 mM and 100 mM EtOH groups as compared to the *Kp* control (0 mM EtOH+*Kp*). At the 48 h time point, we observed up to 17% (R.F.U. 3.38±0.12) and 20% (R.F.U. 3.22±0.11) suppression of proteasome activity in the 50 mM and 100 mM ethanol groups respectively *vs* the *Kp* control (R.F.U. 4.04±0.059) (Fig. 1C). Similar to Fig. 1B, MG132-treated *Kp*-stimulated RAW 264.7 cells showed suppressed proteasome activity in comparison to their respective controls at 24 h and 48 h time points (Fig. 1D). These results revealed that ethanol has a suppressive effect on the chymotrypsin-like activity of the 20S proteasome in both unstimulated and stimulated RAW 264.7 cells.

### Ethanol does not Alter Steady-state Protein Levels of Constitutive Proteasome Subunit Beta 5 (PSMB5) but Suppresses Steady-state Protein Levels of Immunoproteasome Subunit LMP7

**Figure 2:**
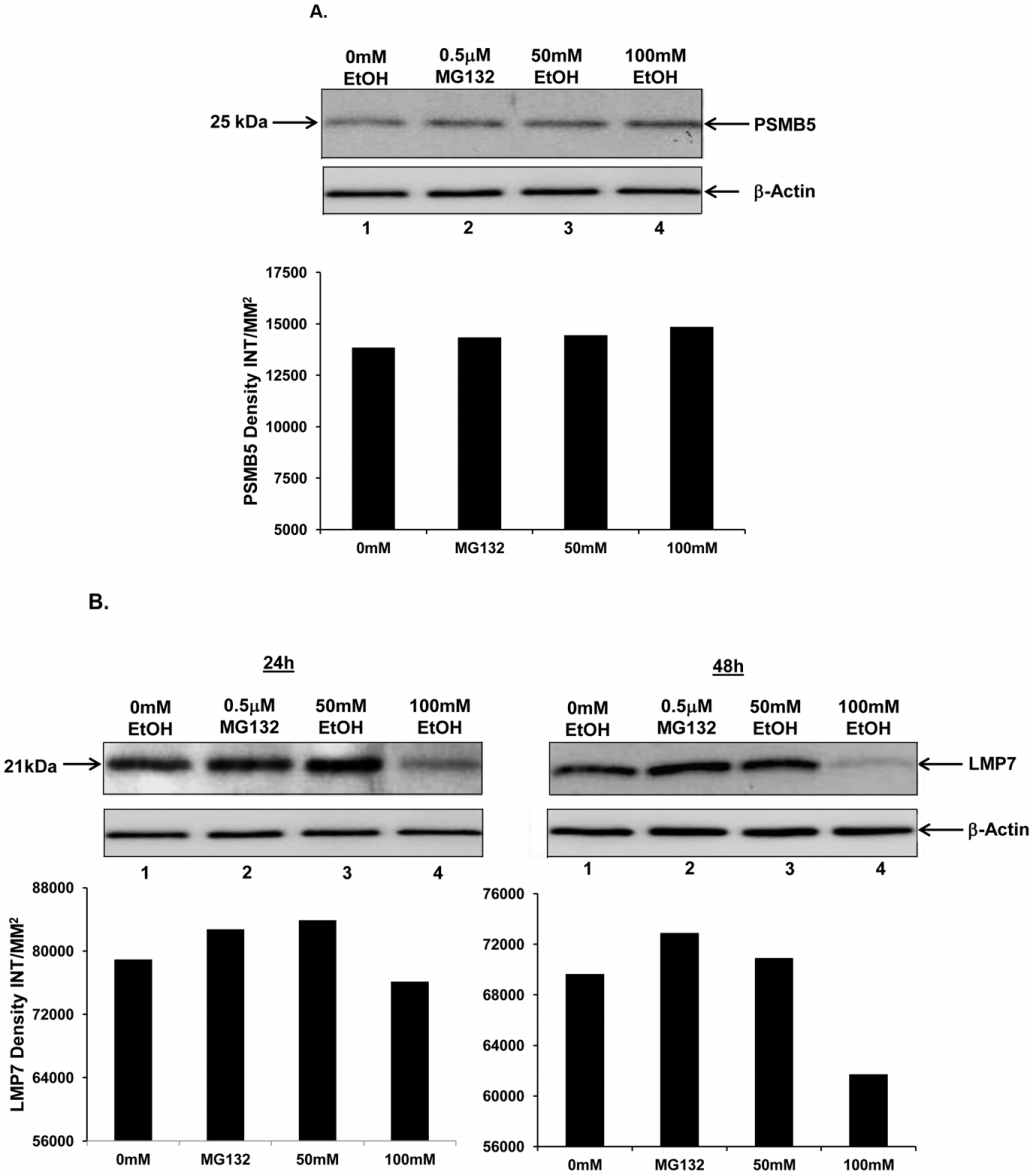
Ethanol does not alter steady-state protein levels of constitutive proteasome subunit beta 5 (PSMB5), but suppresses steady-state protein levels of immunoproteasome subunit LMP7. **A.** Murine unstimulated RAW 264.7 cells (2×10^5^ cells/ml) were treated with 50 mM (lane 3) or 100 mM (Lane 4) ethanol (EtOH) for 48 h. Cells were also treated with MG132 (0.5 uM) (lane 2) as a positive control of proteasome inhibition. 0 mM (control) cells received no treatment (lane1). Cell lysates were obtained 48 h post EtOH exposure. The lysates of triplicate samples were pooled for gel loading, analyzed by 15% SDS-PAGE and immunoblotted with anti-PSMB5 antibody as described in the methods (upper panel). The membrane was stripped and reprobed with anti-beta-actin antibody to ensure equal protein loading as described in the methods section (lower panel). The bar graph represents densitometry quantitation of PSMB5 levels between treatment groups and is represented as INT/mm^2^. The depicted gel blot is representative of two independent experiments. Each band of the blot represents the pooled lysates from three independently treated samples. **B.** Unstimulated RAW 264.7 cells (2×10^5^ cells/ml) were treated with 50 mM (lane 3) or 100 mM (lane 4) EtOH for 24 h (Left panel) or 48 h (Right Panel). Cells were also treated with MG132 (0.5 uM) (lanes 2) as a positive control of proteasome inhibition. 0 mM (control) cells received no treatment (lane1). Cell lysates were obtained 24 h or 48 h post-EtOH exposure. The lysates of triplicate samples were pooled for gel loading, analyzed by 15% SDS-PAGE and immunoblotted with anti-LMP7 antibody as described in the methods (24 h and 48 h upper panels). The membrane was stripped and reprobed with anti-beta-actin antibody to ensure equal protein loading as described in the methods section (24 h and 48 h lower panel). The bar graphs beneath each immunoblot represent densitometry quantitation of LMP7 levels between treatment groups at 24 h and 48 h respectively and is represented as INT/mm^2^. The depicted gel blots are representative of two independent experiments. Each band of the blot represents the pooled lysates from three independently treated samples.

The beta 5 subunit of the proteasome harbors chymotryptic-like activity [13], [24], [25]. Ethanol mediated suppression of proteasome Cht-L activity (Fig. 1) could be either due to inhibition of enzymatic activity or due to suppressed protein expression of the proteasome subunit beta 5. To test the latter possibility, the steady state levels of the constitutive proteasome beta 5 subunit (PSMB5), in ethanol-treated RAW 264.7 cell lysates were assessed by immunoblot analysis using anti-PSMB5 antibody. The lysates of triplicate samples were pooled for gel loading. The depicted gel blot is representative of two independent experiments. As shown in Fig. 2A, upper panel, PSMB5 protein levels remained unaltered in ethanol-treated groups as compared to the untreated controls. The same membrane, as shown in the upper panel, was stripped and reprobed with anti-beta-actin to ensure equal protein loading (Fig. 2, lower panel). The bar graph shows band intensities of PSMB5 shown in the upper panel, as measured by densitometry. These results revealed that ethanol does not alter protein levels of the constitutive proteasome chymotryptic beta 5 subunit.

Macrophages (and other cells of hematopoietic lineage) also simultaneously express a variant form of the proteasome, the immunoproteasome [26]. In the immunoproteasome, the LMP7 subunit (beta 5i) harbors Cht-L activity, though its specificity differs from that of its constitutive beta 5 subunit counterpart [15], [16]. It has been documented that the immunoproteasome is highly efficient at generating immunogenic peptides for antigen presentation [17], [18]. Ethanol did not alter the steady state levels of the beta 5 subunit of the constitutive proteasome (Fig. 2, panel A). To test if ethanol alters the protein expression of the LMP7 subunit of the immunoproteasome, a subunit analogous to the beta 5 constitutive proteasome subunit, we assessed the steady state levels of LMP7 in ethanol-treated RAW 264.7 cell lysates by Western blot analysis using anti-LMP7 specific antibody. The lysates of triplicate samples were pooled for gel loading. The depicted gel is representative of two independent experiments. As shown in Fig. 2, treatment of macrophages with 100 mM ethanol suppressed steady state levels of immunoproteasome subunit LMP7 at both 24 h (Fig. 2B, left panel, lane 4) and 48 h (Fig. 2B, right panel, lane 4). The same membrane, as shown in the upper panel, was stripped and reprobed with anti- beta-actin to ensure equal protein loading (Fig. 2 A and B, lower panels). Bar graphs below each respective blot indicate band intensities of immunoproteasome subunit LMP7 protein levels at the indicated time points. We did not observe any significant change in the levels of LMP7 in cells treated with 50 mM ethanol at the same time points (Fig. 2B, left and right panels, lane 3). Similar to LMP7, ethanol also suppressed protein levels of LMP2 subunit of the immunoproteasome (Fig. S1). These results revealed that ethanol has a suppressive effect on protein levels of the immunoproteasome subunits LMP7 (beta 5i) and LMP2 in RAW 264.7 cells.

### Ethanol Suppresses the 20S Chymotrypsin-like Proteasome in Primary Peritoneal Macrophages

Data obtained using primary cells is more physiologically relevant in comparison to cell line data. Thus, to confirm our cell line results of ethanol induced proteasome suppression, we evaluated the effect of 50 mM and 100 mM ethanol, at 24 h and 48 h, on proteasome activity in primary peritoneal macrophages obtained by peritoneal lavage as described in the Methods section. As before, control (0 mM) cells received no treatment.

**Figure 3:**
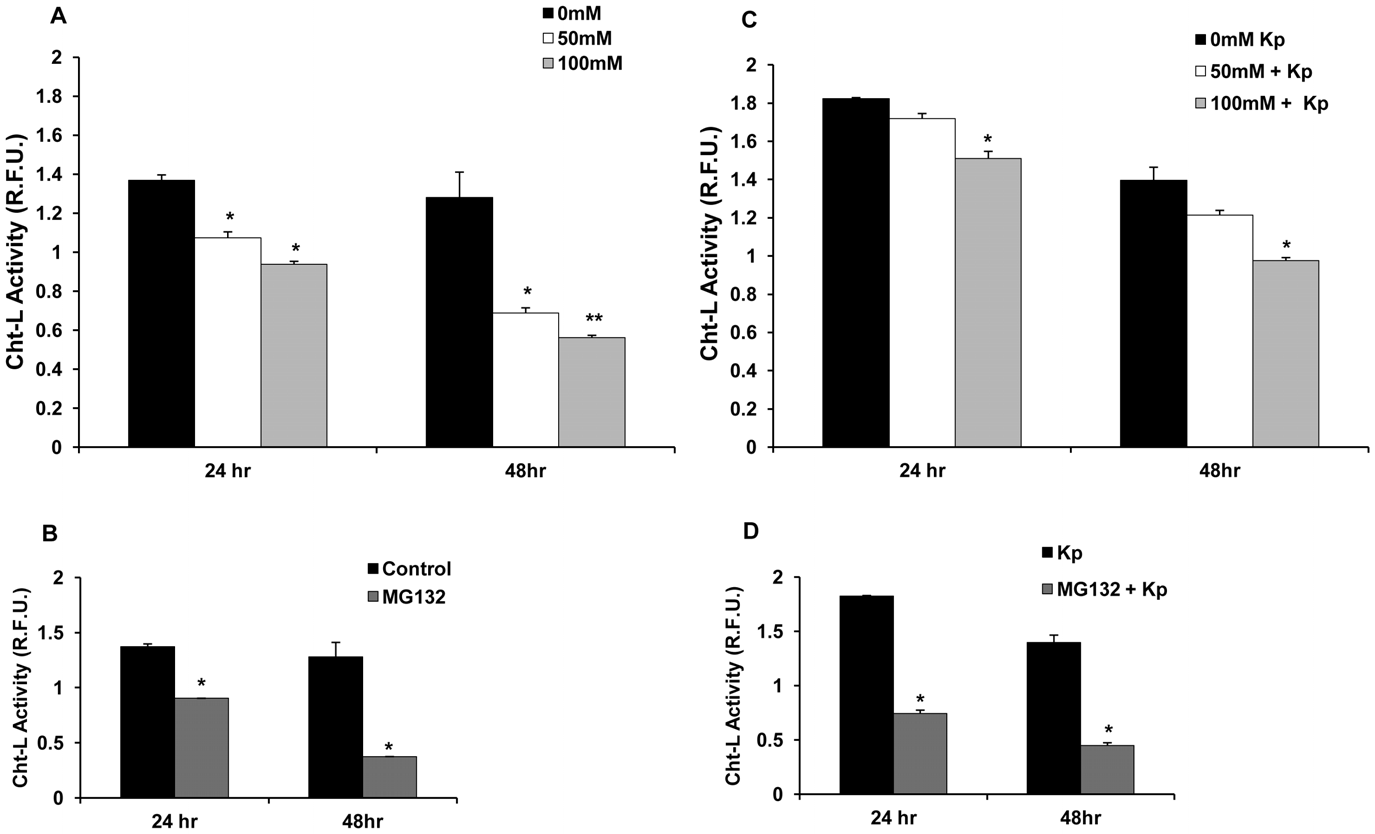
Ethanol suppresses 20S Cht-L proteasome activity in murine primary peritoneal macrophages. A. Murine peritoneal macrophages (2.5×10^5^ cells/ml) were treated with 50 mM or 100 mM EtOH for 24 h or 48 h. 0 mM (control) cells received no treatment. Cell lysates obtained at the indicated time points were analyzed for 20S Cht-L proteasome activity by fluorigenic assay as described in Methods. **B.** Peritoneal macrophages were treated with MG132 (5 uM), as a positive control of proteasome suppression. Cell lysates were obtained at the indicated time points and analyzed as described in Panel A. **C.** Ethanol-treated peritoneal macrophages were stimulated with heat-killed *Kp*, 6 hours after initial EtOH exposure. Cell lysates obtained at indicated time points were analyzed for proteasome activity as described in panel A. **D.** MG132-treated peritoneal macrophages were stimulated with *Kp* as described in Panel C. Cell lysates were obtained at the indicated time points and analyzed for proteasome activity as in Panel A. Proteasome activity data plotted as mean relative fluorescence units (R.F.U.) ± SEM (three replicates per experiment) and is representative of three independent experiments. Higher R.F.U. values indicate higher proteasome activity. *p<0.05 vs. control at the respective time point. **p<0.05 vs. control and 50 mM EtOH at the respective time points.

As shown in Fig. 3A, ethanol significantly suppressed proteasome activity in primary peritoneal macrophages. At 24 h, a significant suppression in Cht-L proteasome activity of up to 22% (R.F.U. 1.07±0.03) or 32% (R.F.U. 0.94±0.02) was observed in peritoneal macrophages exposed to 50 mM or 100 mM EtOH respectively as compared to untreated (0 mM) peritoneal macrophages (R.F.U. 1.37±0.03). Similarly, at 48 h, suppression in Cht-L proteasome activity of up to 46% (R.F.U. 0.69±0.03) or 56% (R.F.U. 0.56±0.01) was observed in the peritoneal macrophages exposed to 50 mM or 100 mM EtOH as compared to the untreated group (R.F.U. 1.28±0.13). Treatment of the peritoneal macrophages with MG132 (5 uM) suppressed proteasome activity by up to 34% (R.F.U. 0.90) and 71% (R.F.U. 0.37) at 24 h and 48 h respectively as compared to the respective untreated controls (24 h R.F.U. 1.37±0.03 and 48 h R.F.U. 1.28±0.13) at those time points (Fig. 3B).

To evaluate if ethanol suppressed 20S Cht-L proteasome activity in stimulated peritoneal macrophages, ethanol-treated peritoneal macrophages were stimulated with heat-killed, *Klebsiella pneumoniae* (*Kp*) (MOI 10∶1) as described in the Methods section. Analysis of cell lysates at the 24 h time point showed suppression in proteasome activity of up to 5.7% (R.F.U. 1.72±0.03) and 17.23% (R.F.U. 1.51±0.04) in the 50 mM and 100 mM EtOH groups as compared to the *Kp*-treated control group (R.F.U. 1.83±0.01) (Fig. 3C). At the 48 h time point, up to 13% (R.F.U. 1.21±0.03) and 30% (R.F.U. 0.98±0.03) suppression of proteasome activity in the 50 mM and 100 mM ethanol groups respectively was observed versus the *Kp*-stimulated 0 mM/control group (R.F.U. 1.40±0.07) (Fig. 3C). Similar to Fig. 3B, MG132-treated *Kp*-stimulated RAW 264.7 cells showed significantly suppressed proteasome activity in comparison to their respective controls at 24 h and 48 h time points (Fig 3D). Viability of the macrophages was unaffected by ethanol treatment or *Kp* stimulation as confirmed by trypan blue staining and microscopy (Data not shown). These results indicated that similar to the cell line results ethanol also suppressed proteasome Cht-L activity in unstimulated and stimulated primary peritoneal macrophages.

### Ethanol Suppresses Ovalbumin Processing and Presentation by Peritoneal Macrophages

**Figure 4:**
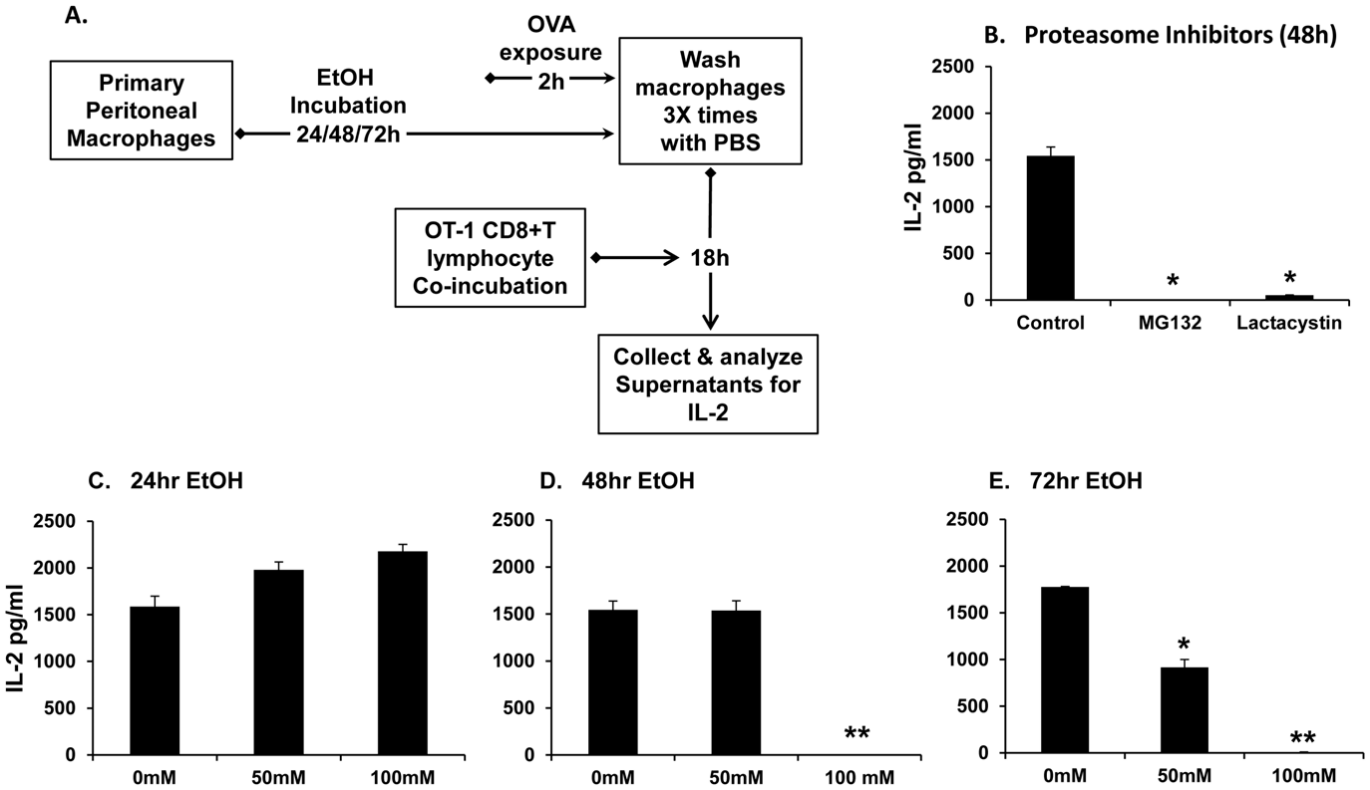
Ovalbumin antigen MHC-Class I processing and presentation is suppressed in ethanol-treated murine primary peritoneal macrophages. **A.** Schematic representation of experimental setup as described in Methods. Briefly, murine peritoneal macrophages were treated 50 mM or 100 mM EtOH for 24, 48 or 72 h. Proteasome inhibitors MG132 (5 uM) or lactacystin (10 uM) were used as positive controls of proteasome inhibition. EtOH-exposed cells were treated with full-length chicken ovalbumin (2 mg/ml) for 2 hours. Macrophages washed with PBS and co-incubated with OT-1 mouse CD8+ cytotoxic T lymphocytes (Ratio 2∶1) for 18 h. Cell supernatants were collected and analyzed for cytokine IL-2. **B.** CD8+ T cell derived IL-2 levels from proteasome inhibitor-treated peritoneal macrophage groups is shown. **C.** CD8+ T cell derived IL-2 levels from 24 h EtOH-treated peritoneal macrophage groups is shown. **D.** CD8+ T cell derived IL-2 levels from 48 h EtOH-treated peritoneal macrophage groups is shown. **E.** CD8+ T cell derived IL-2 levels from 72 h EtOH-treated peritoneal macrophage groups. Data plotted is mean levels of cytokine IL-2 (pg/ml) ± SEM (three replicates per experiment) and is representative of three independent experiments. *p<0.05 vs. control (0 mM) at respective time point. **p<0.05 vs. control (0 mM) and 50 mM EtOH at respective time point.

It has been well demonstrated that constitutive and immunoproteasomes are exclusively involved in the initial steps of processing of antigens into high-affinity peptides for MHC Class I antigen presentation [17], [18]. Since ethanol suppressed proteasome chymotrypsin-like activity in peritoneal macrophages, we examined whether the suppression of chymotrypsin-like activity decrease the proteasome-mediated proteolysis of antigenic protein and consequent MHC Class I -restricted antigen presentation. To study this, ethanol-treated murine primary peritoneal macrophages were exposed to full length chicken ovalbumin (OVA) followed by co-incubation with CD8+ T cells enriched from OT-1 mice (Transgenic mice in which the T cell receptor was designed to recognize ovalbumin residues 257–264, SIINFEKL, in the context of K^b^ [24]). OVA would have to be processed and presented as SIINFEKL-H2K^b^ complex on the surface of the macrophages. The presence of the peptide-K^b^ complexes on the surface of these macrophages would stimulate OVA-K^b^ specific, CD8+ T cells to produce Interleukin 2 (IL-2) cytokine. Measurement of this cytokine was used as a means to measure antigen processing and presentation by the macrophages as described in the Methods section. The experimental procedure is represented in Fig. 4A.

To test the validity of our antigen presentation assay, we examined the processing of OVA in peritoneal macrophages treated with proteasome inhibitors MG132 and lactacystin. As shown in Fig. 4B, as expected, IL-2 production by CD8+ T cells was significantly decreased in peritoneal macrophages incubated with the proteasome inhibitors. These results established that inhibition of the proteasome inhibited presentation of ovalbumin protein thereby confirming the validity of our assay.

In the ethanol-treated macrophages, we observed that at 24 h, processing of ovalbumin by peritoneal macrophages was unaffected by ethanol treatment (Fig. 4C). By the 48 h time point, we observed a significant suppression of IL-2 in the 100 mM EtOH group, as compared to the 0 mM group (1545 pm/ml) (Fig. 4D). Furthermore, by 72 hours, we observed a dose-dependent suppression in IL-2 levels of up to 48% (917 pg/ml) and 98% (10 pg/ml) in the 50 mM and 100 mM groups respectively, as compared to the 0 mM group (1777 pg/ml) (Fig. 4E). The unchanged levels of IL-2 in the 0 mM groups at the various time points indicated efficient processing and presentation of ovalbumin in these cells. IL-2 levels were undetectable in groups of a) macrophages without co-incubation with CD8+ T cells; b) CD8+ T cells alone; c) unstimulated macrophages (without OVA stimulation) and CD8+ T cells; and d) CD8+ T cells incubated with OVA (data not shown). Together our data indicated that ethanol suppressed processing of full length ovalbumin by peritoneal macrophages and subsequently decreased presentation to the CD8+ T cells.

### Ethanol Suppression of the Proteasome Inhibits Processing and Presentation of C-terminal Extended Ovalbumin Peptide

The proteolytic activities of constitutive and immunoproteasomes are mainly involved in the C-terminal processing of antigens thereby giving rise to N-terminal extended peptides [11]. These N-extended peptides are then further processed by aminopeptidases in the cytoplasm and ER before being complexed to MHC Class I for presentation [11]. In order to correlate ethanol induced proteasome suppression with impaired antigen processing, we utilized C-terminal extended OVA peptide (SIINFEKLTE) in our study since the C-terminal extension (amino acids Threonine (T) and Glutamic acid (E)) of the peptide would need to undergo proteasome cleavage to generate the SIINFEKL epitope for MHC Class I presentation [11]. Because proteasome activity is essential in generating antigenic peptides with the precise C-terminus, and because ethanol suppressed proteasome activity (Fig. 3) and OVA presentation (Fig. 4), we wanted to determine if ethanol would impair the processing and presentation of SIINFEKLTE by the peritoneal macrophages and consequently attenuate IL-2 production by CD8+ T cells. The experimental setup is similar to Fig. 4A except that C-terminal extended OVA peptide, SIINTEKLTE was used instead of full length OVA.

**Figure 5:**
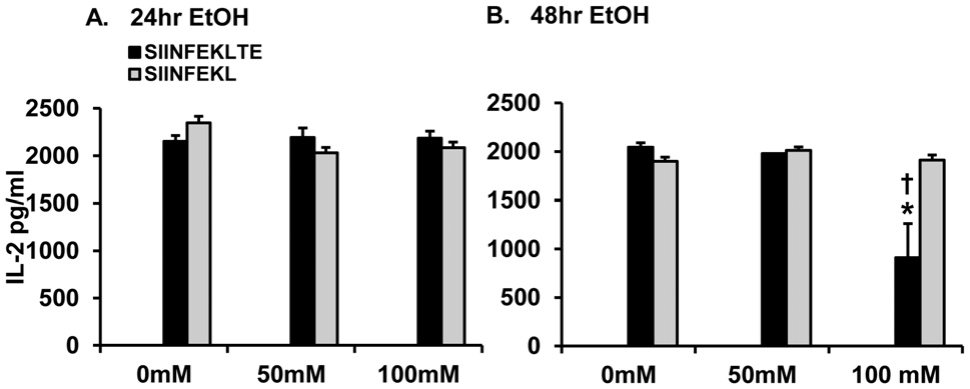
Antigen presentation of C-terminal extended SIINFEKLTE is suppressed in ethanol-treated murine primary peritoneal macrophages. Experimental setup is the same as detailed in Fig. 4A, except that instead of full length OVA the C-terminal extended SIINTEKLTE peptide was used. **A.** CD8+ T cell-derived IL-2 levels from 24 h EtOH-treated peritoneal macrophage groups is shown. **B.** CD8+ T cell-derived IL-2 levels from 48 h EtOH-treated macrophage groups is shown. Data plotted is mean levels of cytokine IL-2 (pg/ml) ± SEM (three replicates per experiment) and is representative of three independent experiments. **p<0.05 vs. control and 50 mM EtOH at respective time point.

As shown in Fig. 5, the processing and presentation of SIINFEKLTE remained unaffected in peritoneal macrophages exposed to 50 mM ethanol for 24 h (panel A) and 48 h (panel B) as indicated by the IL-2 levels which were similar to the 0 mM groups at the respective time points. In the 100 mM ethanol exposed groups, at 24 h, ethanol exposure did not have an effect on processing and presentation of SIINFEKLTE (panel A). However at 48 h ethanol exposure, the processing and presentation of SIINFEKLTE was suppressed by as much as 55% (911 pg/ml) (panel B,) as compared to the 0 mM (2044 pg/ml) SIINFEKLTE-exposed groups at the same time point (panel B). In contrast, SIINFEKL peptide without C-terminal extension was efficiently presented by these cells both at 24 h (panel A) and 48 h (panel B) of ethanol exposure. These results using SIINFKL vs. SIINFKLTE peptides revealed the requirement of the proteasome in the processing and presentation of the C-terminally extended SIINFEKLTE. Similar to the previous experiment, IL-2 levels were undetectable in groups of a) macrophages without co-incubation with CD8+ T cells; b) CD8+ T cells alone; c) unstimulated macrophages (No SIINFEKLTE stimulation) and CD8+ T cells; and d) CD8+ T cells incubated with OVA (data not shown). Additionally, the levels of IL-2 from the 0 mM groups exposed to SIINFEKLTE (Fig. 5, panels A and B,) were similar to the levels of IL-2 from the 0 mM groups exposed to full length OVA (Fig. 4, panels C and D) indicating that processing and presentation of the full length protein and the C-terminal extended peptide occurred in a relatively similar manner in the macrophages. These results together with the results shown in Figs. 1 and 3 revealed that ethanol suppresses proteasome function and consequently MHC Class I antigen processing and presentation in peritoneal macrophages.

## Discussion

Mounting evidence reveals that binge-drinking suppresses multiple aspects of the innate and adaptive immune system. As a result, alcoholics have an increased susceptibility to infections. Alcohol interferes with the functions of many cellular and molecular components that are part of the immune system [3], [6], [27]. In this study, we investigated the effects of acute ethanol exposure on the proteasome activity and peptide-processing machinery of murine peritoneal macrophages. We demonstrate that ethanol suppresses the proteasome function consequently impairs peptide processing required for MHC Class I restricted antigen presentation in macrophages.

Macrophages simultaneously express both the constitutive proteasome and immunoproteasome [26]. In the current study, using an *in-vitro* model of acute ethanol exposure, we have demonstrated that ethanol suppresses >90% of the proteasome Cht-L activity suggesting that ethanol suppresses Cht-L activities of both the c20S and i20S proteasome. Additionally, ethanol suppressed the steady-state levels of LMP7 (i20S subunit harboring Cht-L activity) and not PSMB5 (c20S subunit harboring Cht-L activity) in macrophages. Together our results point towards the possibility that, although ethanol suppresses the Cht-L activity of both proteasome and immunoproteasome, immunosuppressive effect of ethanol may majorly be due to the suppression of the Cht-L activity associated with the immunoproteasome. Further experiments are needed to confirm whether the immunosuppressive effect of ethanol is indeed due to the Cht-L activity associated with the immunoproteasome. We do not know if ethanol suppresses the trypsin-like (β2/β2i) and caspase-like (β1/β1i) activities of the c20S and i20S proteasome. However, we did not notice any apparent changes in levels of polyubiquitylated protein substrates in the ethanol-treated samples as compared to the untreated groups (data not shown), suggesting that ethanol does not suppress the overall function of proteasome in macrophages. Our findings that ethanol suppresses proteasome function are in concurrence with prior studies in hepatocytes [20], [21], [28] and monocyte-derived macrophages with respect to the suppressive effects of ethanol on proteasome activity [26]. Ethanol-mediated oxidative stress as well as by-products of ethanol metabolism, such as acetaldehyde suppress proteasome function in hepatocytes and monocyte derived macrophages though further experiments would be needed to confirm this in our model [20], [21], [26], [28].

Interestingly, although, ethanol suppressed >90% of the proteasome Cht-L activity in unstimulated macrophage cells, only 30% of the Cht-L activity was suppressed in ethanol-treated Kp-stimulated macrophages under the same conditions (100 mM for 48 hrs) (Figs. 1 and 3). On the other hand, MG132 completely suppressed Cht-L activity in both unstimulated and Kp-stimulated macrophages. The reason for this variable suppression of the Cht-L activity in MG132 and ethanol-treated unstimulated vs. stimulated macrophages is unclear. However, it has been previously demonstrated that ethanol (25 mM) protects chymotrypsin-like activity of the proteasome in HIV-1 infected but not in uninfected cells [26]. These published studies suggest that decreased suppression of the Cht-L activity may be a result of the protective effect of ethanol in Kp-challenged macrophages.

Proteasomes have primarily been associated with recognition and degradation of polyubiquitylated protein substrates within cells, thereby regulating intracellular protein turnover [12], [22], [24], [25]. Additionally, in conjunction with numerous cytosolic and endoplamic reticular (ER) peptidases, both constitutive (c20S) and immunoproteasomes (i20S), contribute to cleavage of antigenic peptides. Prior work indicates that proteasomes are exclusively responsible for processing/trimming MHC Class I epitopes at the carboxy terminus of the antigenic protein leaving only the epitope or, most of the time, an N-terminal extended peptide which is then further trimmed by cytoplasmic or ER aminopeptidases [17], [19]. In macrophages, and other APC’s, antigen presentation represents a critical initial step necessary for the downstream efficient priming and induction of the adaptive immune response. We investigated if ethanol induced proteasome suppression had any effect on antigen processing and presentation of MHC class I antigens. In the current manuscript, we show that ethanol suppresses MHC-class I-mediated antigen presentation in macrophages. Using C-terminal extended OVA peptide SIINFEKLTE we have further demonstrated that the reduced MHC-class I-mediated antigen presentation is due to the ethanol-mediated suppression of the proteasome function in macrophages. Although, however, ethanol has been reported to suppress proteasome function, ethanol-mediated impairment of the antigen presentation was not reported so far. To the best of our knowledge, our data represent the first description of ethanol-induced suppression of the antigen presentation in macrophages.

The effects of ethanol are far more complex and are known to alter numerous host responses at the molecular, cellular and overall system levels. A growing body of evidence indicates that acute ethanol impairs innate immune responses by suppressing cell signaling pathways, proinflammatory cytokine and chemokine production and release, phagocytic and killing capacity of phagocytes, cellular recruitment and antigen presentation [4]–[6], [29]–[31]. The involvement of proteasomes, to some level, in a number of these cellular processes has been established and thus further research would be needed to elucidate how exactly ethanol induced proteasome impairment may alter these cellular functions thereby impairing immune function [18], [24], [32]. The data presented in this study has revealed one of the several plausible mechanisms by which acute ethanol exposure may impair cellular functions in macrophages thereby disrupting innate immunity and downstream adaptive immunity.

In conclusion, the present study shows that acute ethanol exposure suppresses proteasome chymotrypsin-like enzymatic activity as well as protein levels of immunoproteasome subunit LMP7. This ethanol-induced impairment of proteasome and immunoproteasome function led to the suppression of antigen processing of ovalbumin and ovalbumin-related peptides leading to suppressed MHC Class I antigen presentation to CD8+ T cells. These results provide an important mechanism to explain the immunosuppressive effects of acute ethanol exposure.

## Supporting Information

Figure S1 **Ethanol suppresses steady-state protein levels of immunoproteasome subunit LMP2.** Unstimulated RAW 264.7 cells (2×10^5^ cells/ml) were treated with 50 mM (lane 2) or 100 mM (lane 3) EtOH for 24 h (left panel) or 48 h (Right Panel). 0 mM (control) cells received no treatment (Lane1). Cell lysates were obtained 24 h or 48 h post-EtOH exposure. The lysates of triplicate samples were pooled for gel loading, analyzed by 15% SDS-PAGE and immunoblotted with anti-LMP2 antibody as described in the methods (24 h and 48 h upper panels). The membrane was stripped and reprobed with anti-beta-actin antibody to ensure equal protein loading as described in the methods section (24 h and 48 h lower panel). The bar graphs beneath each immunoblot represent densitometry quantitation of LMP2 levels between treatment groups at 24 h and 48 h respectively and are represented as INT/mm^2^. The depicted gel blots are representative of two independent experiments.
(TIF)
