# Supplementary figures and images for: Suppression of the Macrophage Proteasome by Ethanol Impairs MHC Class I Antigen Processing and Presentation
Source: PLoS One. 2013 Feb 25;8(2):e56890. doi: 10.1371/journal.pone.0056890 (PMC3581560; doi:10.1371/journal.pone.0056890)

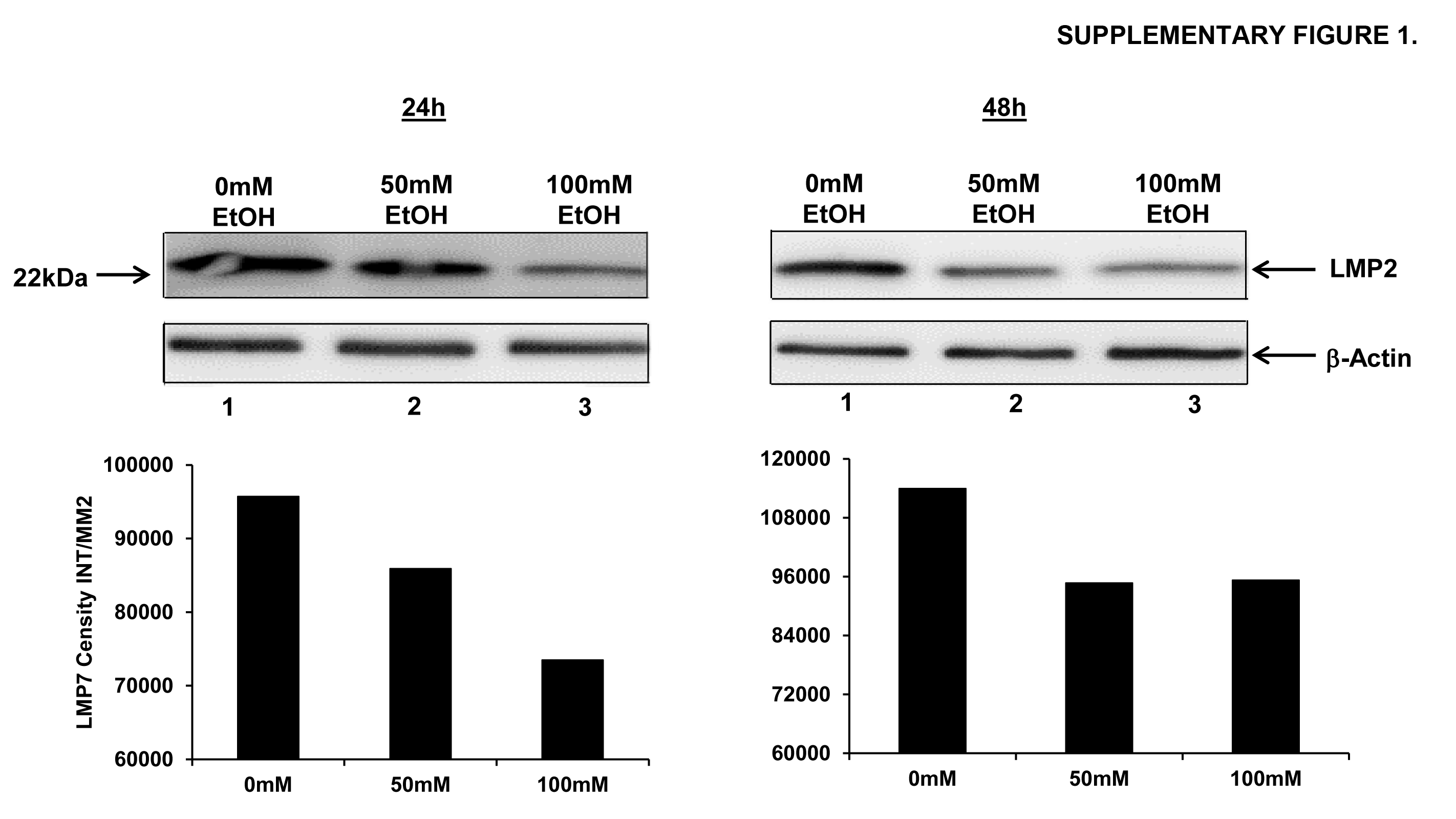

Supplement: Figure S1 — Ethanol suppresses steady-state protein levels of immunoproteasome subunit LMP2. Unstimulated RAW 264.7 cells (2×10^5^ cells/ml) were treated with 50 mM (lane 2) or 100 mM (lane 3) EtOH for 24 h (left panel) or 48 h (Right Panel). 0 mM (control) cells received no treatment (Lane1). Cell lysates were obtained 24 h or 48 h post-EtOH exposure. The lysates of triplicate samples were pooled for gel loading, analyzed by 15% SDS-PAGE and immunoblotted with anti-LMP2 antibody as described in the methods (24 h and 48 h upper panels). The membrane was stripped and reprobed with anti-beta-actin antibody to ensure equal protein loading as described in the methods section (24 h and 48 h lower panel). The bar graphs beneath each immunoblot represent densitometry quantitation of LMP2 levels between treatment groups at 24 h and 48 h respectively and are represented as INT/mm2. The depicted gel blots are representative of two independent experiments. (TIF) [file pone.0056890.s001.tif]
